# Stage-specific functional networks associated with cognitive impairment in Parkinson's disease: a pilot fNIRS study

**DOI:** 10.3389/fnagi.2025.1562203

**Published:** 2025-05-13

**Authors:** Jiarui Zhao, Yulai Gong, Zhenfang Lin, Jie Yang, Jiahuan Zou, Xia He, Yongsheng He

**Affiliations:** ^1^School of Medical and Life Sciences, Chengdu University of Traditional Chinese Medicine, Chengdu, Sichuan, China; ^2^Department of Neurology, Affiliated Sichuan Provincial Rehabilitation Hospital of the Chengdu University of Traditional Chinese Medicine, Chengdu, Sichuan, China; ^3^Department of Neurology, First Affiliated Hospital of Jiaxing University, Jiaxing, Zhejiang, China; ^4^Department of Neurosurgery, Sichuan Academy of Medical Sciences & Sichuan Provincial People's Hospital, Chengdu, Sichuan, China

**Keywords:** Parkinson's disease, functional near-infrared spectroscopy, cortical activation, functional connectivity, cognitive impairment

## Abstract

**Objective:**

This study aimed to investigate cortical activation and functional connectivity during Stroop task performance in Parkinson's disease (PD) using functional near-infrared spectroscopy (fNIRS).

**Methods:**

Forty-five individuals with PD and fourteen healthy controls completed neuropsychological assessments and underwent fNIRS scanning while performing the Stroop task. PD participants were categorized into normal cognition (PD-NC, *n* = 6), mild cognitive impairment (PD-MCI, *n* = 22), and dementia (PDD, *n* = 17) groups. *Z* scores were calculated across cognitive domains, including attention, working memory, executive function, language, memory, and visuospatial function.

**Results:**

During the Stroop task, significant hypoactivation in the dorsolateral prefrontal cortex (DLPFC), primary motor cortex (M1), and premotor cortex (PMC) were observed in the PD-MCI group, while PDD patients showed increased activation in the medial prefrontal cortex (mPFC), orbitofrontal cortex (OFC), and dorsolateral prefrontal cortex (DLPFC). Increased activation in DLPFC was significantly correlated with poorer executive function outcomes. Functional connectivity analysis revealed that both PD-NC and PD-MCI groups had significantly enhanced interhemispheric connectivity compared to healthy controls, with pronounced interhemispheric connectivity in PD-MCI. In contrast, the PDD group exhibited reduced connectivity among the premotor cortex (PMC), ventrolateral prefrontal cortex (VLPFC), and orbitofrontal cortex (OFC), compared to the PD-MCI group.

**Conclusion:**

While PD-MCI patients showed reduced cortical activation relative to PDD, they exhibited extensive connectivity across cortical regions, suggesting an expanded cortical network as compensation. In PDD, the mPFC, left OFC, and left DLPFC displayed the highest cortical activation and alongside reduced functional connectivity, which may reflect widespread atrophy across multiple brain regions. These findings highlight fNIRS as a potential tool for characterizing cognitive impairment stages in PD.

## 1 Introduction

Parkinson's disease (PD) is traditionally recognized as a neurodegenerative disorder typically characterized by motor symptoms. However, an increasing body of research highlights the importance of non-motor symptoms (NMS), such as cognitive disorder, depression, and olfaction dysfunction, in the presymptomatic stages of PD (Irwin et al., 2013; Tolosa et al., 2021). Among these disorders, cognitive impairment has emerged as a major contributor to reduced quality of life and early disability in PD patients (Orgeta et al., 2020), with 19–42.5% of PD patients exhibiting mild cognitive impairment (PD-MCI) at the time of diagnosis (Yarnall et al., 2014). PD-MCI represents an intermediate phase between normal cognition and PD dementia (PDD), characterized by measurable cognitive decline without substantial functional impairment in daily life (Bai et al., 2022). Understanding the relationship between cognitive states and the underlying mechanisms in PD is essential for advancing early diagnostic strategies and identifying therapeutic targets to reduce cognitive decline.

Despite significant advances in PD research, the pathophysiological mechanisms underlying cognitive impairment in PD remain poorly understood. Previous research has suggested that alterations in neurotransmitters levels (e.g., HVA, 5-HT, GABA, and Ach), decreased amyloid-β (Aβ) and αSyn, and increased neurofilament light (NFL) levels are significant contributors of PD-MCI (Paolini Paoletti et al., 2023; Liu et al., 2020). In addition, variations in specific genes have been identified as significant risk factors for cognitive impairment in PD (Mollenhauer et al., 2014). Neuroimaging findings have further revealed structural and functional brain alterations associated with PD-related cognitive impairment, including chronic cerebral hypoperfusion, gray matter atrophy, and white matter abnormalities within the corpus callosum (Chen et al., 2016; Bledsoe et al., 2018; Jia et al., 2019; Pierzchlińska et al., 2021). Understanding these pathological mechanisms is essential for identifying biomarkers and developing targeted interventions to mitigate cognitive decline in PD.

A growing area of PD research focuses on alterations in functional brain network to elucidate how neuropathological changes affect behavioral outcomes in PD (Gao, 2016). The nervous system integrates information through coordinated activity across various brain regions, highlighting the importance of functional network dynamics in maintaining cognitive and motor functions (Sakoğlu, 2011). Functional connectivity (FC) analysis has become a powerful method for probing these dynamics, providing insights into interregional interactions, potential biomarkers, and neurophysiological changes underlying PD-related cognitive impairment. Recent studies have identified FC disruptions in PD-MCI, particularly within the default mode network (DMN), visual network, and sensorimotor network (SMN) (Hou, 2021). Specifically, PD-MCI patients exhibited increased posterior cingulate cortex (PCC) connectivity, whereas reduced PCC connectivity was observed in PDD patients (Zhan et al., 2018). Additionally, working memory deficits are associated with both PD-MCI and PDD, with stronger responses during 0-back and 1-back tasks compared to healthy controls (Hattori et al., 2022). Furthermore, previous fMRI studies have shown that memory impairments in PD-MCI patients are associated with both intra- and inter-network disruptions, including reduced FC within the SMN and decreased connectivity between the SMN and attention-related networks, such as the dorsal attention network (DAN), ventral attention network (VAN), and frontoparietal network (FPN) (Delgado-Alvarado et al., 2023). Despite these advances, the progression of PD-related cognitive impairment and its underlying neural mechanisms remain poorly understood.

Functional near-infrared spectroscopy (fNIRS) is a non-invasive neuroimaging technique that measures changes in brain tissue concentration of oxygen-hemoglobin ([Oxy-Hb]) and deoxygen-hemoglobin ([Deoxy-Hb]) associated with neuronal activation (Whelan, 2020). By capturing these hemodynamic signals, fNIRS can dynamically monitor brain activity, revealing neural functions and regional synchronization, particularly in capturing stage-specific neural networks across different task phases (Pinti et al., 2020; Shu et al., 2024). Compared to functional magnetic resonance imaging (fMRI), fNIRS offers several advantages, including cost-effectiveness, portability, and greater tolerance for motion artifacts (Ferrari and Quaresima, 2012). While fMRI requires patients to remain still, which is limiting for individuals with motor disorders, especially those with PD. Therefore, fNIRS is particularly well-suited for studying complex cognitive and motor functions in clinical settings (Ayaz et al., 2013; Gawryluk, 2017).

Recent years have seen a growing interest in the application of fNIRS in PD research, particularly in exploring gait disorders, cognitive impairments, and rehabilitation strategies (Bonilauri et al., 2022; Kvist, 2024). While neuropsychological assessments, such as the Clock Drawing Test (CDT), are commonly employed to evaluate executive function, visuospatial processing, and semantic memory in PD, and it indicates that the activation pattern of the PFC is a key indicator for identifying PD-related cognitive decline (Schejter-Margalit et al., 2022). However, the role of the parietal lobe in cognitive impairment in PD cannot be ignored. Studies have shown that abnormalities in the functional connectivity of the anterior parietal lobe are closely associated with cognitive impairments in PD patients, particularly affecting higher-order cognitive functions (Yeager et al., 2024). Therefore, in our study, we specifically focused on the prefrontal cortex (PFC), sensorimotor cortex (SMC), and premotor and supplementary motor areas (PMC/SMA), as these regions are crucial for uncovering the cognitive mechanisms in PD (Behrmann et al., 2004; Alvarez and Emory, 2006; Souza-Couto et al., 2023).

Although current research have focused on cognitive changes in PD patients, most studies focus on isolated cognitive stages (Guo et al., 2021; Inguanzo et al., 2021), failing to capture the progressive changes in cognitive function across different stages. This limitation presents challenges for early detection and intervention strategies, particularly in the early identification of PD-MCI. Furthermore, current neuroimaging techniques, especially resting-state neuroimaging techniques may be insufficient for capturing task-related functional changes associated with cognitive decline. Therefore, our study aims to address this gap by systematically evaluating the differences in neural activity across various subgroups of PD-related cognitive impairment.

Executive function impairments, including deficits in attention, working memory, processing speed, and impulsivity, often emerge in early-stage PD (Dirnberger and Jahanshahi, 2013). The Stroop task, a widely used measure of executive function and inhibitory control, employs both incongruent and congruent color/word stimuli (Stroop-type) to provide a robust assessment of cognitive control deficits in PD (Rabin et al., 2005; Faulkner et al., 2021). Recent evidence has shown that fNIRS-based Stroop task measurements, when integrated with a support vector machine (SVM) classifier, can distinguish PD-MCI patients from cognitively normal individuals with up to 83.3% accuracy (Shu et al., 2024). We used fNIRS combined with the Stroop task to assess neural activity differences across different PD subgroups. We hypothesize that there are significant differences in cortical activation and functional connectivity patterns between PD subgroups (PD-NC, PD-MCI, PDD) and healthy controls (HCs). By comparing these differences, we sought to identify shared and distinct hemodynamic characteristics across cognitive states. Our results would enhance our understanding of the neural mechanisms underlying PD-related cognitive impairment, with implications for early diagnosis, personalized interventions, and predicting disease progression.

## 2 Methods

### 2.1 Participants

Forty-five patients with PD were recruited from Sichuan Bayi Rehabilitation Center and classified into three cognitive subgroups: PD-NC (*n* = 6), PD-MCI (*n* = 22), and PDD (*n* = 17). In addition, fourteen HCs with no history of cognitive or neurological impairments were recruited from the community. PD diagnoses were confirmed by movement disorder specialists based on the UK PD Brain Bank Criteria. Exclusion criteria included the presence of other neurodegenerative disorders (e.g., Alzheimer's disease, frontotemporal dementia), cognitive impairment due to non-neurological conditions, major psychiatric illness, severe systemic diseases, or a history of cerebral surgery. The HCs were screened to confirm normal cognitive function, with Mini-Mental State Examination (MMSE) scores ≥28 and Montreal Cognitive Assessment (MOCA) scores ≥26. The research protocol received approval from the Institutional Review Board of Sichuan Bayi Rehabilitation Center, and all participants provided written informed consent before participation.

### 2.2 Clinical and cognitive evaluations

Clinical assessments collected demographic data, medication usage, and PD-specific measures, including the Hoehn & Yahr (H-Y) stage (Goetz et al., 2008) and the MDS-UPDRS (Anon, 2003), focusing on motor function (UPDRS-III) in the “ON” state. Levodopa equivalent daily dose (LEDD) was calculated (Tomlinson et al., 2010). Neuropsychological evaluations included the MMSE, MOCA (Folstein et al., 1975; Nasreddine et al., 2005), and a battery of cognitive tests assessing five domains: attention/working memory, executive function, language, memory, and visuospatial abilities, following MDS Task Force recommendations (see Supplementary Table 1). Composite z-scores were calculated for each domain based on normative data, normalizing scores to a common distribution. Cognitive classifications into PD-NC, PD-MCI and PDD adhered to MDS Task Force criteria (Emre et al., 2007; Litvan et al., 2013). Participants not meeting PD-MCI or PDD criteria were classified as PD-NC.

### 2.3 Experimental procedure

Participants were seated comfortably in front of a computer screen while wearing an fNIRS cap, ensuring a stable fit and accurate positioning. Prior to the formal experiment, each participant received instructions and completed a practice session to ensure familiarization with the Stroop task. Once comprehension was confirmed, participants performed the Stroop task during a 510-s experimental session comprising three test cycles. Each cycle included a 140-s Stroop test phase followed by a 30-s rest period. Task performance, including reaction times and accuracy, was recorded for analysis.

### 2.4 Data acquisition

**Figure 1:**
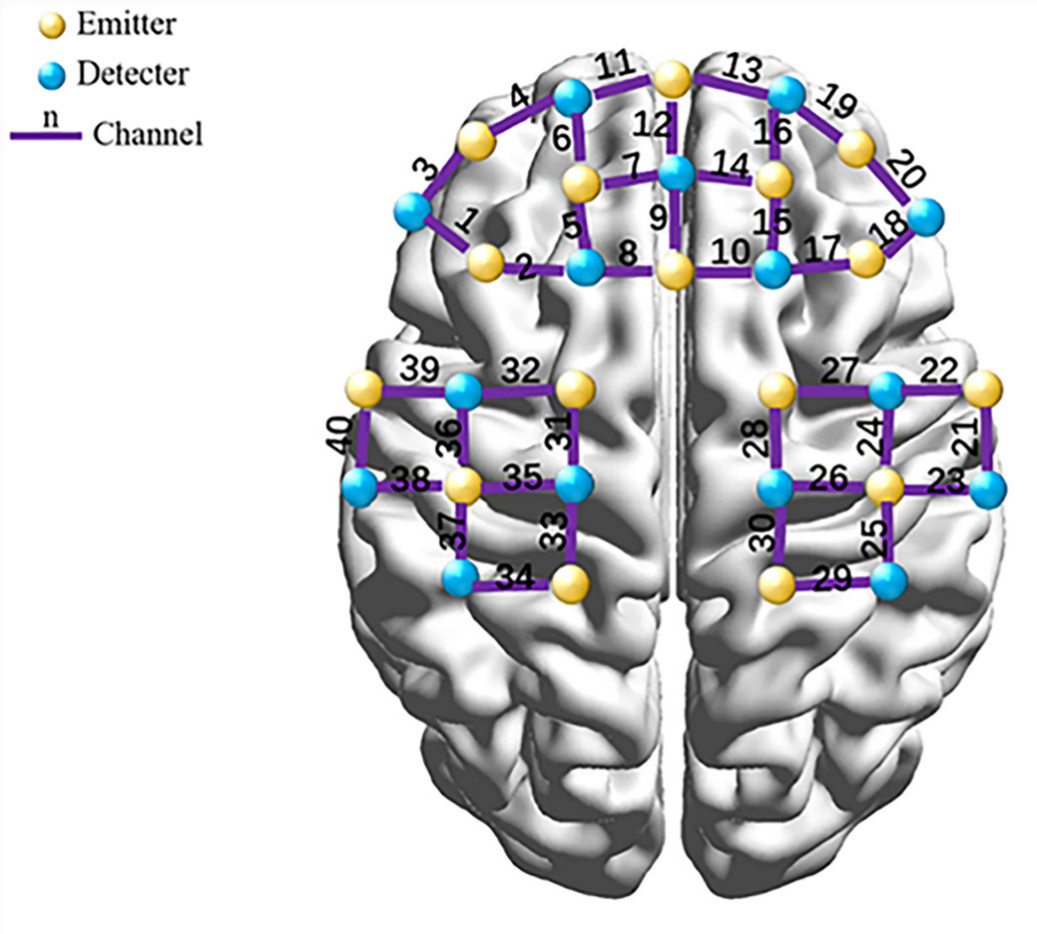
Configuration of fNIRS channels.

Functional brain activity was recorded using a multi-channel fNIRS system (NIRSport, NIRx Medical Technologies LLC) with a sampling rate of 7.8 Hz. Hemodynamic changes in oxyhemoglobin (HbO), deoxyhemoglobin (HbR), and total hemoglobin (HbT) were measured in the frontal and parietal lobes, and chromophore concentrations were calculated using the Modified Beer-Lambert Law (MBLL). The optode array consisted of 16 emitters and 15 detectors, arranged into 31 probes and 40 measurement channels, targeting the prefrontal cortex (PFC), sensorimotor cortex (SMC), and premotor and supplementary motor areas (PMC/SMA) following the international 10–20 EEG system. Emitters and detectors were spaced 3 cm apart, with the central optode positioned at FPz and the lowest probes aligned along the Fp1-Fp2 line (Figure 1).

### 2.5 Data preprocessing

fNIRS data preprocessing was conducted using the Homer2 package in MATLAB (2022b). Raw light intensity data were first converted to optical density and subsequently processed into hemoglobin concentration changes using MBLL with a differential path-length factor of [6 6]. Motion artifacts were detected and corrected using the motion artifact detection tool. A bandpass filter (0.01–0.1 Hz) was applied to reduce noise related to physiological signals such as respiration and cardiac pulsation. Functional connectivity was analyzed by calculating Pearson's correlation coefficients between all channel pairs, providing a network-based perspective on cortical activation and inter-regional communication during task performance.

### 2.6 Data analyses

The Shapiro-Wilk test was used to assess the normality of clinical and cognitive data distributions. Group comparisons for demographic data were conducted using one-way analysis of variance (ANOVA) for parametric data and Kruskal-Wallis tests for non-parametric data in SPSS (version 25). *Post hoc* comparisons were performed with Bonferroni correction to adjust for multiple comparisons. To identify underlying cognitive patterns, principal component analysis (PCA) was conducted based on normalized neuropsychological variables. PCA was performed using a correlation matrix, with Varimax rotation applied to enhance interpretability by optimizing variable loadings. The cognition *z* scores in healthy control, PD subgroups were compared using one-way analysis of covariance (ANCOVA), and Bonferroni correction was used for post-test comparisons of between group differences. Age and education level were adjusted as covariates during the ANCOVA. The Stroop task performance (accuracy and response time) was evaluated using a two-factor repeated-measures ANOVA, with factors of (congruent vs. incongruent) task and group (PD-NC, PD-MCI, PDD, and HC). fNIRS data were processed and analyzed using MATLAB (2022b, MathWorks, Inc.). Hemodynamic changes in HbO were used as the primary metric for assessing cortical activation. Group differences in HbO levels were analyzed using one-way ANOVA, with false discovery rate (FDR) correction applied to control for multiple comparisons. For *post hoc* analysis, Bonferroni correction was employed to ensure robust statistical inference. FC was assessed by calculating the Pearson correlation coefficients (*r*) between Hbo time-series data from different ROIs, with the coefficient ranging from −1 to 1. The connectivity strength as follows: *r* < 0.5 indicated low FC, 0.5 ≤ *r* < 0.7 indicated moderate FC, while *r* ≥ 0.7 represented strong FC (Cohen, 2009; Chen et al., 2022). Independent sample *t*-tests were used to compare connectivity strength between groups.

To investigate the relationship between task-related HbO concentrations, MMSE scores, and task performance accuracy, Pearson's correlation analysis was performed. Statistical significance was set at *p* < 0.05 for all tests. Visualization of results, including group differences and correlation trends, was generated using GraphPad Prism (version 9).

## 3 Results

### 3.1 Participant characteristics

Table 1 presents the demographic and clinical characteristics of the participants. No significant differences were found between the groups in age or gender distribution. However, the PD-NC group had significantly more years of education than both PD-MCI and PDD groups (*p* < 0.05). No significant differences in disease duration or H-Y scores were found within the PD subgroups. Motor function, assessed by MDS-UPDRS Part III, showed significant differences, with PDD showing worse motor function than PD-NC, and PD-MCI showing poorer performance than PD-NC. Dopamine agonist dosage was higher in the PDD group.

**Table 1:** Demographic and clinical characteristics.

**Characteristics**	**HC (*n* = 14)**	**PD–NC (*n* = 6)**	**PD–MCI (*n* = 22)**	**PDD (*n* = 17)**	***p*–value**
Age	60.36 ± 5.26	63.33 ± 13.140	66.14 ± 8.871	68.35 ± 8.86	0.057
Gender (male/female)	8/6(57.1/42.9)	3/3 (50/50)	11/11 (50/50)	10/7(58.8/41.2)	0.941
Education	9 (9–12.75)	14 (9–16.25)	9 (6–12.00)	6 (2–6.50)	<0.001^acdef^
PD duration	–	7.5 (3.5–11.00)	5 (2.75–8.00)	7 (4–11)	0.468
Hoehn and Yahr stage	–	2 (1–2.125)	2 (1.75–2.125)	2 (2–2.5)	0.253
UPDRS Part III score	–	14.00 ± 5.514	22.27 ± 6.311	26.18 ± 6.12	<0.001^ade^
Levodopa equivalent doses	–	673.04 ± 325.52	567.7386 ± 257.14	851.41 ± 307.97	0.014^af^
MMSE scores	29.5 (29.0–30.0)	29 (28–29)	28 (27.75–29.25)	23 (22–24.5)	<0.001^acef^
MOCA scores	27 (26–28)	26.5 (26–27.5)	22 (21–24)	19 (14.5–19.5)	<0.001^abcdef^
Memory *z* score	1.23 ± 0.67	0.24 ± 0.74	−0.10 ± 0.51	−0.97 ± 0.62	<0.0005^abcdef^
Language *z* score	0.74 ± 0.75	0.54 ± 0.36	0.07 ± 0.63	−0.90 ± 1.08	<0.0005^acef^
Attention/working memory *z* score	1.07 ± 0.45	0.80 ± 0.39	−0.21 ± 0.68	−0.89 ± 0.82	<0.0005^abcdef^
Executive function *z* score	−0.89 ± 0.63	−0.89 ± 0.48	0.08 ± 0.78	0.94 ± 0.70	<0.0005^abcdef^
Visuospatial *z* score	0.74 ± 0.28	0.74 ± 0.27	0.14 ± 0.44	−1.01 ± 1.21	<0.0005^abce^

Data are expressed as mean (standard deviation). Gender data were analyzed with χ2 test. Other p values were derived from Kruskal Wallis test for non–parametric test and independent one–way ANOVA for parametric test. Data presented as mean ± SD unless noted. PD duration, Hoehn and Yahr stage, MMSE scores and MOCA scores results are reported with Interquartile range. MDS–UPDR, Movement Disorder Society–Unified Parkinson's Disease Rating Scale; MMSE, Mini–Mental State Examination; MoCA, Montreal Cognitive Assessment; NC, normal cognition; PD–MCI, Parkinson's disease with mild cognitive impairment; PDD, Parkinson disease dementia.

^a^*post hoc* comparisons showed no significant differences between HCs and patients with PD–NC.

^b^*post hoc* comparisons showed significant differences between HCs and patients with PD–MCI.

^c^*post hoc* comparisons showed significant differences between HCs and patients with PDD.

^d^*post hoc* comparisons showed significant differences between PD–NC and patients with PD–MCI.

^e^*post hoc* comparisons showed significant differences between PD–NC and patients with PDD.

^f^*post hoc* comparisons showed significant differences between PD–MCI and patients with PDD.

Regarding cognitive performance, the PDD group had significant deficits on the MMSE compared to PD-MCI, PD-NC, and HC groups (*p* < 0.05), while no differences were found between PD-NC and PD-MCI. MoCA scores were significantly lower in the PDD group than in the other groups (*p* < 0.05). PD-NC scored higher than PD-MCI (*p* < 0.001). Covariance analysis was conducted with groups as the independent variable, cognition *z* scores as the dependent variable, and education as a covariate. The results have shown that cognitive domain *z-*scores differed significantly across groups (*p* < 0.0005), with the PDD group showing the most severe impairments. No significant differences were found between PD-MCI and PDD in the visuospatial domain, nor between PD-NC and PD-MCI in language and visuospatial domains. There were no significant differences between PD-NC and HC groups across cognitive domains.

### 3.2 Stroop task performance

**Figure 2:**
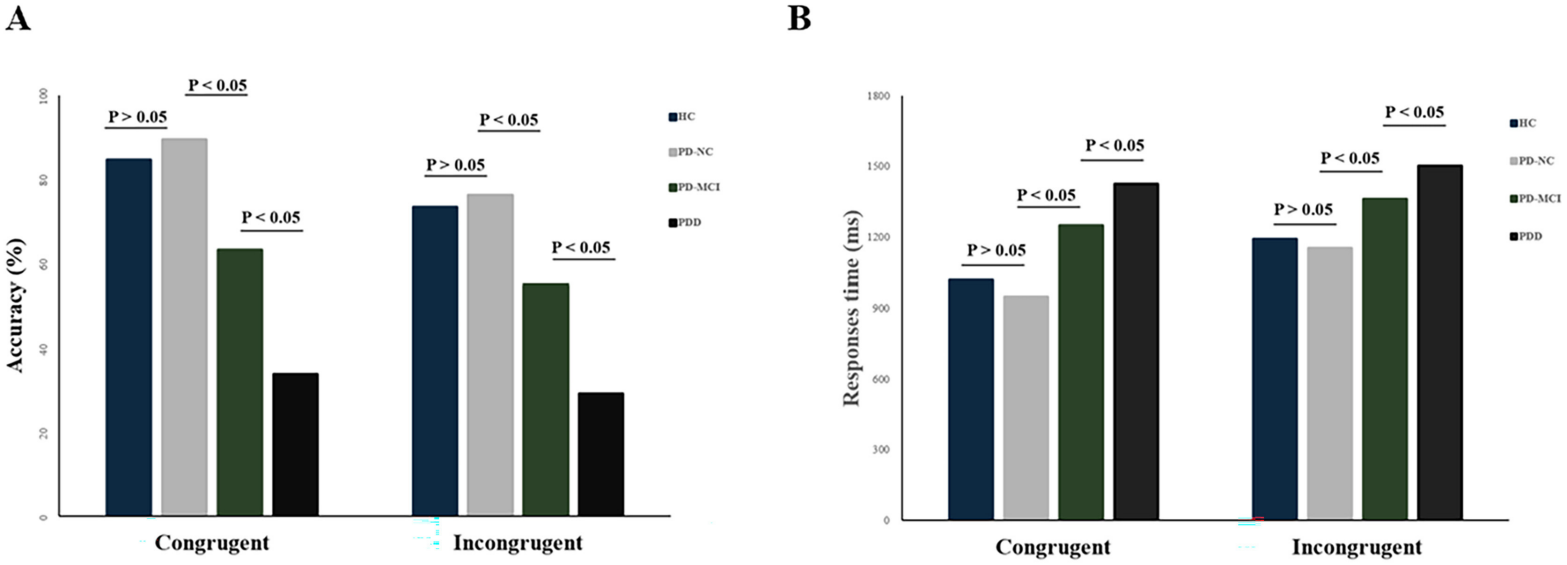
Behavior results for task performance: **(A)** rate of correct answer; **(B)** response times.

A two-way repeated-measures ANOVA (task condition × group) revealed a significant interaction between group and task condition was found (*F* = 3.931, *p* < 0.05). Specifically, the PD-MCI and PDD groups showed significantly slower response times compared to the PD-NC group for both congruent and incongruent trials (congruent tasks: *p* < 0.05; incongruent tasks: *p* < 0.05, *F* = 189.085, η^2^ = 0.07). Similarly, task accuracy differed significantly among the PD subgroups, with the PD-NC group outperforming the PD-MCI and PDD groups under both congruent and incongruent conditions (congruent tasks: *p* < 0.05; incongruent tasks: *p* < 0.05, *F* = 410.632, η^2^ = 0.15). In contrast, no significant differences were observed in either response times or accuracy between the HC and PD-NC groups across both trial types (*p* > 0.05) (Figure 2).

### 3.3 Comparison of hemodynamics response across four groups

**Figure 3:**
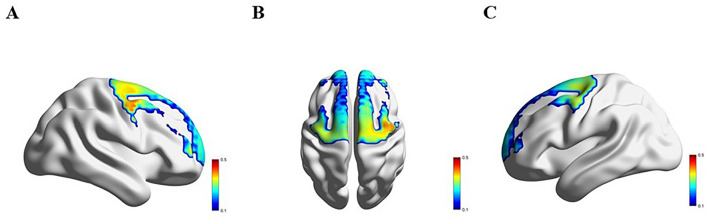
Activation patterns during the Stroop task in the HC group: **(A)** right view; **(B)** superior view; **(C)** left view. Colors represent mean β-values over time, with red indicating higher activation compared to blue regions.

**Figure 4:**
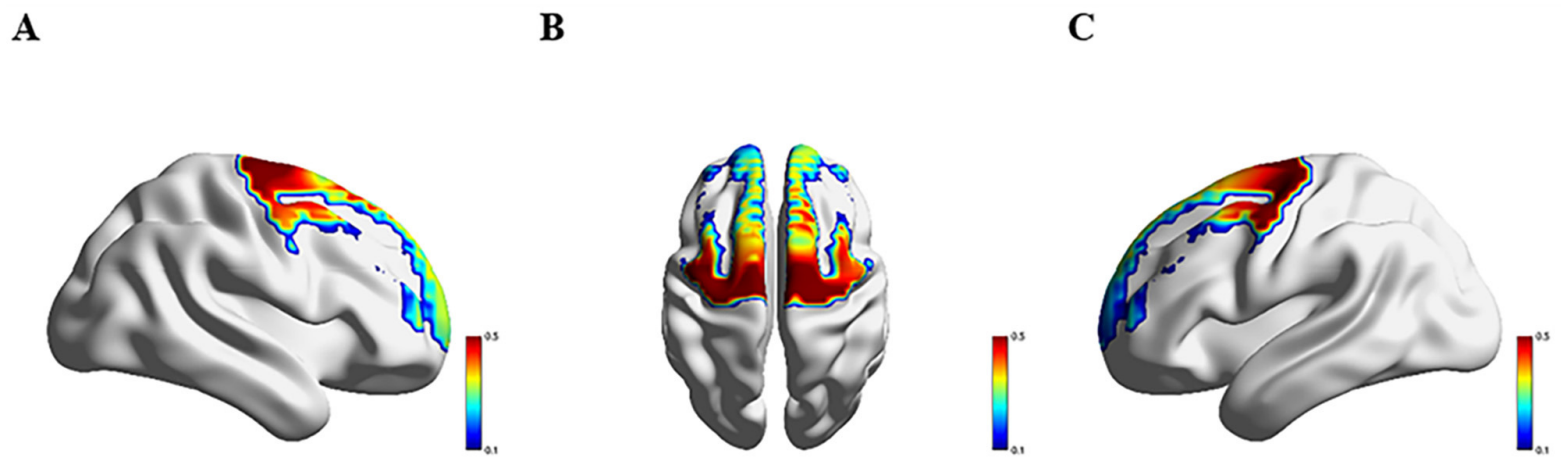
Activation patterns during the Stroop task in the PD-NC group: **(A)** right view; **(B)** superior view; **(C)** left view. Colors represent mean β-values over time, with red indicating higher activation compared to blue regions.

**Figure 5:**
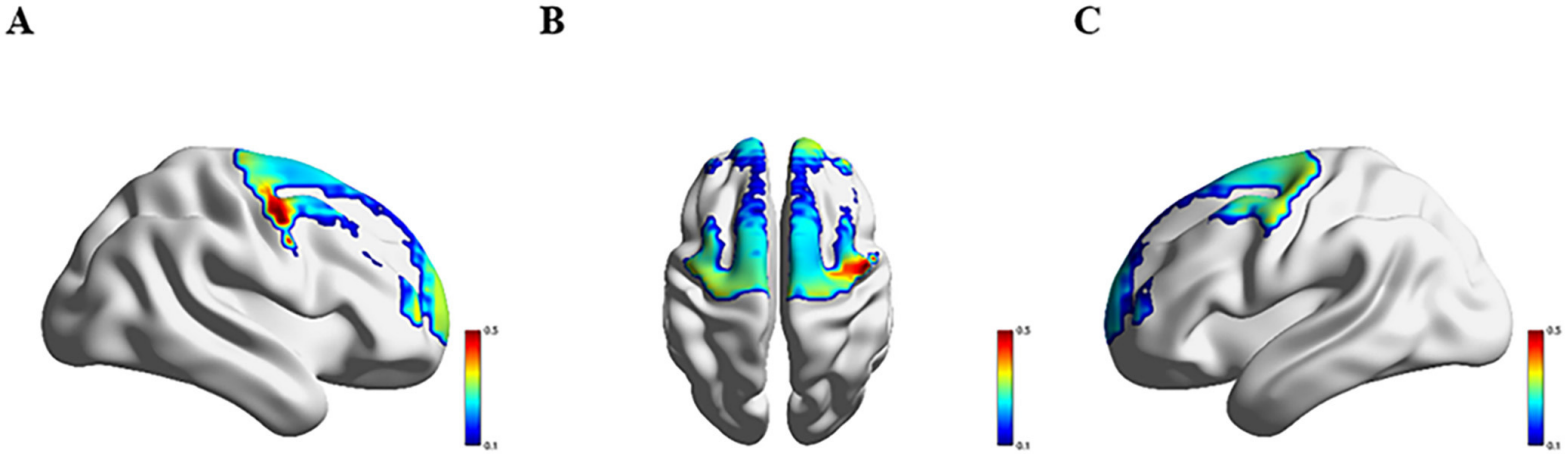
Activation patterns during the Stroop task in the PD-MCI group: **(A)** right view; **(B)** superior view; **(C)** left view. Colors represent mean β-values over time, with red indicating higher activation compared to blue regions.

**Figure 6:**
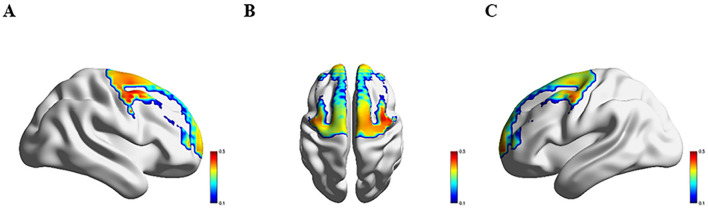
Activation patterns during the Stroop task in the PDD group: **(A)** right view; **(B)** superior view; **(C)** left view. Colors represent mean β-values over time, with red indicating higher activation compared to blue regions.

**Figure 7:**
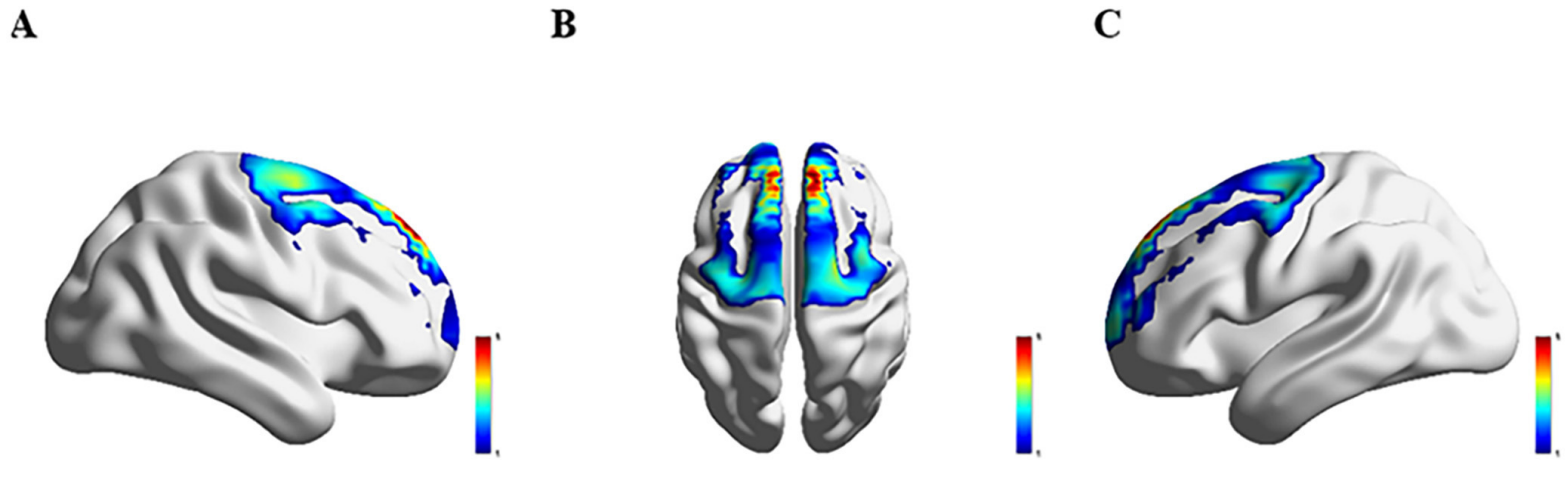
**(A–C)** illustrate the right, superior, and left hemispheres, respectively, depicting the fNIRS channels with significant differences after the FDR correction.

Figures 3–6 show the brain activation intensity and activation patterns during the Stroop task across all PD subgroups (PD-NC, PD-MCI, and PDD) and HCs. All groups exhibited widespread activation in the bilateral PFC and SMC while performing the Stroop task. One-way ANOVA with FDR correction identified significant group differences in HbO levels. As shown in Figure 7, significant activation differences were observed in 13 fNIRS channels (CH1, CH5, CH6, CH8, CH10, CH11, CH12, CH24, CH25, CH26, CH33, CH36, and CH37) within the prefrontal and parietal lobes during the Stroop task. After Bonferroni and permutation-based FDR corrections, 12 channels remained statistically significant (*p* < 0.05), corresponding to the following cortical regions: the right dorsolateral prefrontal cortex (R_DLPFC, CH10), left dorsolateral prefrontal cortex (L_DLPFC, CH5, CH8), left ventrolateral prefrontal cortex (L_VLPFC, CH1), left orbital frontal cortex (L_OFC, CH6, CH11), left premotor cortex (L_PMC, CH36), left primary motor cortex (L_M1, CH33), right primary motor cortex (R_M1, CH26), left primary somatosensory cortex (L_S1, CH37), right primary somatosensory cortex (R_S1, CH25), and medial prefrontal cortex (mPFC, CH12).

Significant differences in Oxy-Hb activation were observed across groups. Compared to HC group, PD-NC group exhibited higher Oxy-Hb activation in the L_VLPFC and L_PMC (*p* < 0.05). Relative to PD-NC group, PD-MCI group showed reduced Oxy-Hb activation in the L_DLPFC, L_PMC, L_M1, and R_M1 (*p* < 0.05 for all). Conversely, PDD group demonstrated higher Oxy-Hb activation compared to PD-MCI group in the L_DLPFC, L_PMC, R_M1, L_OFC, and mPFC (*p* < 0.05 for all). When compared to PD-NC group, PDD group showed elevated Oxy-Hb activation in the L_OFC (*p* < 0.05). Relative to HC group, PDD group exhibited significantly increased Oxy-Hb activation in the L_VLPFC (*p* < 0.05) (Table 2).

**Table 2:** Areas with significant changes in cortical activity between groups during Stroop task.

			***p*** **–value**				
**Brain areas**	**Channel**	**p–value**	**HC vs.PD–NC**	**HC vs.PDD**	**PD–NC vs.PD–MCI**	**PD–NC vs.PDD**	**PD–MCI vs.PDD**
mPFC	CH12	0.006^*^	1	0.085	0.493	1	0.008^*^
L–PMC	CH36	0.015^*^	0.027^*^	0.436	0.044^*^	0.632	0.736
L–M1	CH33	0.200	0.062	1	0.015^*^	0.233	0.967
L–S1	CH37	0.090	–	–	–	–	–
L–DLPFC	CH5, CH8	<0.001^*^	0.109	0.040^*^	0.002^*^	0.892	<0.001^*^
L–VLPFC	CH1	0.010^*^	0.045^*^	0.044^*^	0.258	1	0.444
L–OFC	CH6, CH11	0.009^*^	0.515	0.026^*^	0.948	0.022^*^	0.002^*^
R–DLPFC	CH10	0.234	0.228	0.237	0.127	0.704	0.094
R–M1	CH26	0.005^*^	1	1	0.034^*^	1	0.018^*^
R–S1	CH25	0.420	0.125	0.450	0.465	0.294	0.637

P-valued by Bonferroni *post-hoc* tests and p-value correction.

^*^p < 0.05.

### 3.4 Correlation analysis

**Figure 8:**
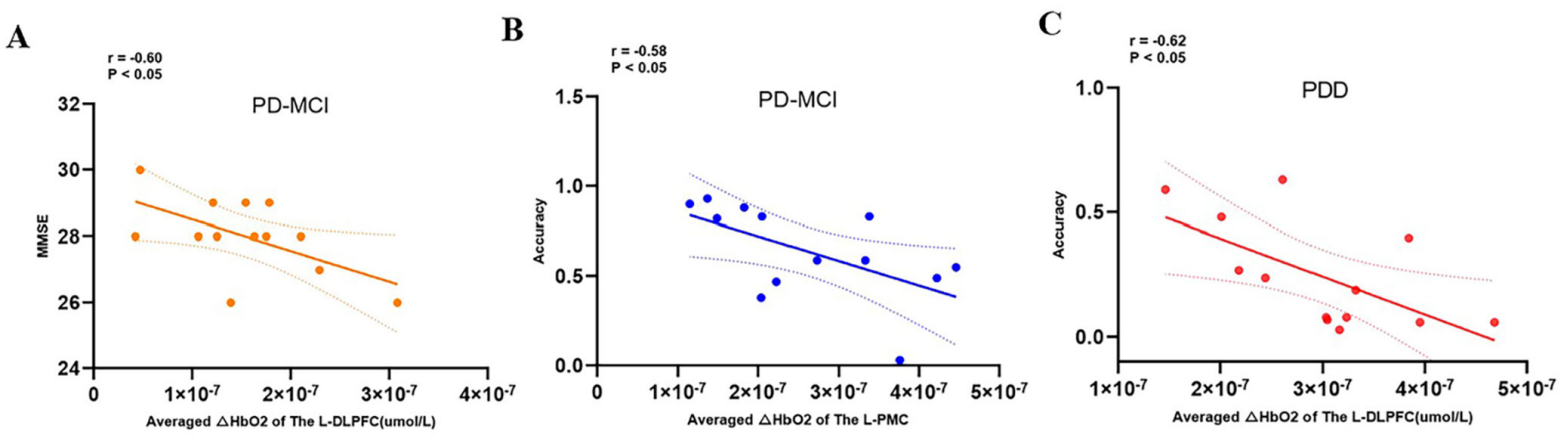
Correlation analysis between the averaged ΔHbO2 and demographic and clinical characteristics. The Averaged ΔHbO2 of the left DLPFC was negatively correlated with the MMSE **(A)** and The Averaged ΔHbO2 of the left PMC was negatively correlated with the accuracy **(B)** The Averaged ΔHbO2 of the left DLPFC was negatively correlated with the accuracy **(C)**.

The results of correlation analyses are illustrated in Figure 8. Among PD-MCI group, the mean ΔHbO concentration in the L_DLPFC exhibited a significant negative correlation with MMSE scores (*r* = −0.60, *p* < 0.05), indicating that greater activation in this region was associated with lower cognitive performance. Additionally, the mean ΔHbO concentration in the L_PMC showed a significant negative correlation with task accuracy (*r* = −0.58, *p* < 0.05). In the PDD group, the mean ΔHbO concentration in the L_DLPFC was significantly negatively correlated with task accuracy (*r* = −0.62, *p* < 0.05).

### 3.5 Functional connectivity characteristics and differences

**Figure 9:**
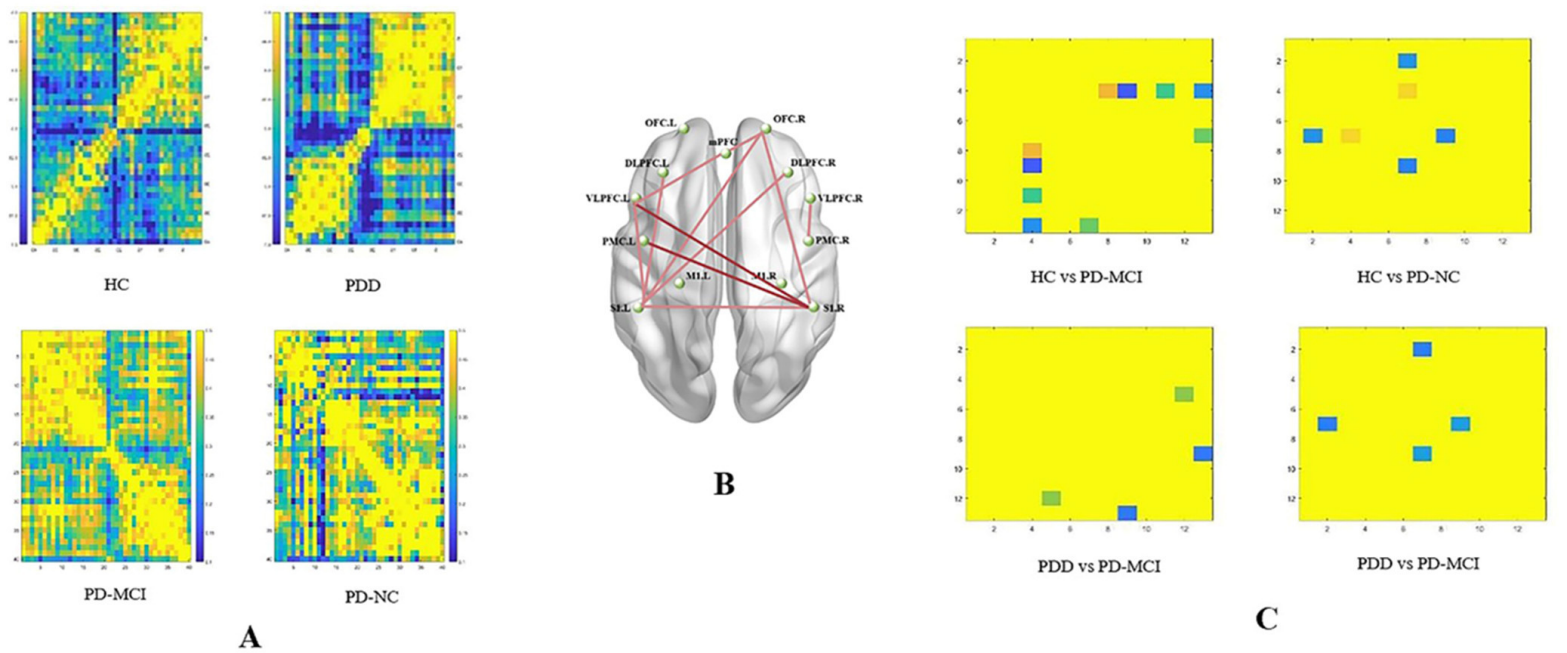
The task state functional connectivity in deoxyhemoglobin. **(A)** Group-averaged task-state functional connectivity matrix diagram. **(B)** ROI-based connections with significant inter-group differences (*p* < 0.05, FDR corrected). **(C)** The results of *post-hoc* comparisons (*p* < 0.05, Bonferroni corrected). The color bar indicates the statistical significance threshold.

Group-specific FC patterns during task performance are presented in Figure 9. While overall FC differences across the HC, PD-NC, PD-MCI, and PDD groups were not statistically significant (*p* > 0.05, FDR-corrected), ROI-based analyses identified significant group differences in specific interregional connections. These connections included S1.L-VLPFC.L (*p* = 0.009), S1.L-DLPFC.L (*p* = 0.040), S1.L-OFC.R (*p* = 0.016), S1.L-DLPFC.R (*p* = 0.029), S1.L-S1.R (*p* = 0.045), S1.R-VLPFC.L (*p* = 0.014), S1.R-PMC.L (*p* = 0.016), S1.R-OFC.R (*p* = 0.032), OFC.R-VLPFC.L (*p* = 0.013), and PMC.R-VLPFC.R (*p* = 0.034) (all FDR-corrected).

*Post-hoc* Bonferroni comparisons revealed significant group differences in FC. The PD-NC and PD-MCI groups demonstrated significantly higher FC compared to the HC group in the following connections: S1.L–DLPFC.L (*r* = 0.525), S1.L–VLPFC.L (*r* = 0.625), S1.L–OFC.R (*r* = 0.503), S1.L–DLPFC.R (*r* = 0.503), S1.L–S1.R (*r* = 0.763), S1.R–OFC.R (*r* = 0.474), S1.R–VLPFC.L (*r* = 0.711), and S1.R–PMC.L (*r* = 0.754) (*p* < 0.05 for all). In contrast, the PDD group exhibited significantly reduced FC compared to the PD-MCI group in OFC.R–VLPFC.L (*r* = 0.389) and PMC.R–VLPFC.R (*r* = 0.669) (*p* < 0.05). Furthermore, the PDD group showed lower FC in S1.R–PMC.L (*r* = 0.382) and S1.R–VLPFC.L (*r* = 0.317) compared to the PD-NC group (*p* < 0.05). No significant FC differences were observed between the PD-MCI and PD-NC groups (Figure 9C).

## 4 Discussion

This fNIRS study investigated brain activation differences among PD patients with varying levels of cognitive impairment (PD-NC, PD-MCI, and PDD) compared to HCs during Stroop task performance. The results revealed distinct and progressive patterns of cortical activation associated with cognitive decline. Specifically, the PD-NC group exhibited significantly greater Oxy-Hb activation in regions linked to cognitive and motor control, including the L_VLPFC and L_PMC, compared to HCs. In contrast, the PD-MCI group exhibited reduced Oxy-Hb activation in areas essential for executive and motor functions, such as the DLPFC, L_PMC, and M1 relative to PD-NC group. These results provide novel evidence for the progressive disruption of brain networks involved in cognitive decline in PD.

The VLPFC plays an important role in working memory, decision-making, goal-directed behavior, cognitive control, and behavioral inhibition (Levy and Wagner, 2012; Zhan et al., 2018). Enhanced activation of the VLPFC observed in PD-NC patients during Stroop task is consistent with prior studies showing increased VLPFC activity during complex cognitive tasks (Pelicioni et al., 2022). Such activation may reflect a compensatory mechanism, enabling PD-NC patients to maintain task performance despite early PD-related neural changes. Similarly, increased activation in the PMC among PD-NC patients may reflect adaptive neural mechanisms responding to the dual motor and cognitive demands of the Stroop task.

The DLPFC and M1 are crucially involved in executive control, response inhibition, and motor sequencing (Ridding et al., 1995). Consistent with previous studies showing improved cognitive function through repetitive transcranial magnetic stimulation (rTMS) in these regions (Khedr et al., 2020), our findings further emphasize their roles in managing cognitive impairment in PD. Notably, the decreased activation of the DLPFC, M1, and PMC in PD-MCI patients may indicate dysfunction in the cortical-basal ganglia-thalamic (CBT) circuits, which are associated with dopaminergic deficits in the striatum (Chung et al., 2018) and basal ganglia (Bhattacharjee et al., 2020). Dysfunctional interactions among these regions likely impair the ability to manage executive demands and inhibitory control during cognitively challenging tasks, contributing to reduced Stroop task performance in PD-MCI patients.

Interestingly, PDD patients exhibited overactivation of the DLPFC compared to PD-MCI patients. This increased activation may reflect compensatory recruitment of cognitive resources triggered by extensive neural degeneration. However, these compensatory mechanisms appear inadequate for improving behavioral performance. Specifically, our results revealed a negative correlation between Oxy-Hb activation in the DLPFC and Stroop task accuracy among PDD patients, suggesting that the observed overactivation represents neural inefficiency. As cognitive impairment advances in PD, executive control, attention, and inhibitory mechanisms degrade substantially, particularly affecting performance during complex incongruent trials. Thus, increased prefrontal activation in PDD likely represent an ineffective compensatory strategy associated with the advanced stage of cognitive decline, reflecting a complex interplay between degenerative processes and attempted neural compensation.

Previous research has identified two primary functional networks that underlie brain activity: the task-positive network activated during cognitive tasks, and the DMN predominantly active at rest (Yang et al., 2018). The Stroop task involves both cognitive and motor demands, activating several key brain regions such as S1, which plays a critical role in processing sensory input and ensuring timely responses. In this study, PD-NC patients exhibited significantly increased functional connectivity between S1 and executive and motor regions (PMC and VLPFC) compared to healthy controls. S1 and PMC are critical nodes of the sensorimotor network (SMN), involved in motor planning and control, while the VLPFC is a core region of the executive control network (ECN), supporting higher cognitive functions. Therefore, the enhanced connectivity between S1, PMC, and VLPFC in PD-NC patients may reflect compensatory adaptations in response to early cognitive and motor function declines. By enhancing SMN-ECN interactions, PD-NC patients may preserve task performance, offsetting early neural changes linked to cognitive and motor impairments (Filippi et al., 2020; Kunst et al., 2018).

Neuropathological research has demonstrated selective α-synuclein pathology affecting the fronto-limbic circuitry (Braak et al., 2002), notably involving regions such as S1, DLPFC, VLPFC, and OFC (Saliasi et al., 2014), which are critically associated with cognitive deficits (Wang et al., 2019). Neuroimaging studies have further revealed structural and functional declines in these areas in PD-MCI patients, including reduced metabolism (Wang et al., 2017) and cortical atrophy (Luks et al., 2010). Despite these degenerative changes, our results showed enhanced functional connectivity among S1, DLPFC, VLPFC, and OFC in PD-MCI patients. This finding supports the notion that increased inter-hemispheric functional connectivity may represent compensatory plasticity responses to disrupted structural connectivity (Matsui et al., 2007), notably involving corpus callosum alterations (Agosta et al., 2013). Increased connectivity between sensory and executive regions in PD-MCI patients may reflect adaptive integration strategies designed to sustain cognitive function, however, albeit with limited effectiveness.

Importantly, we observed a seemingly paradoxical finding in the PD-MCI group, wherein reduced activation in the left PMC was positively associated with task accuracy. This result implies potential resource reallocation toward the frontoparietal network (FPN), including regions such as DLPFC, VLPFC, and OFC (Kazemi et al., 2018; Li et al., 2018), to prioritize cognitive demands. Such a rapid redistribution of neural resources aligns with previous hypotheses regarding compensatory cognitive strategies in MCI (Jilka et al., 2014). Additionally, the involvement of the OFC, a critical node of the salience network (SN), likely optimizes cognitive control by selectively prioritizing relevant stimuli and actions (Baggio and Junqué, 2019; Aracil-Bolaños et al., 2019). Nevertheless, despite enhanced compensatory functional connectivity, PD-MCI patients demonstrated more pronounced executive dysfunction compared to PD-NC individuals, underscoring the limitations of these compensatory mechanisms. Persistent executive deficits likely reflect underlying dysfunction within the bilateral insula and anterior cingulate cortex, key regions implicated in cognitive control and executive processing (Christopher et al., 2015).

In PDD patients, earlier compensatory FC adaptations decline due to widespread neurodegenerative processes. Specifically, PDD patients exhibited significantly reduced connectivity between prefrontal and parietal regions, reflecting impaired cortical information integration (Fiorenzato et al., 2024). Reduced frontoparietal interactions directly parallel clinical manifestations of PDD, such as profound deficits in executive function, attention, and memory. Furthermore, disruptions within critical cognitive networks including the ECN and DMN have been documented in PDD (Fiorenzato et al., 2024), consistent with localized coherence loss and bilateral frontal atrophy (Borroni et al., 2015) as well as corpus callosum volume reduction (Goldman et al., 2017). Thus, our findings suggest that observed connectivity and activation patterns in PDD may reflect diminished compensatory capacities coupled with structural and functional deterioration underlying advanced cognitive impairment in PD.

## 5 Limitations

This study has several limitations that must be acknowledged. First, the small sample size of the PD-NC group (*n* = 6) limited statistical power, potentially affecting the stability and reproducibility of the findings. Moreover, the overall sample size was insufficient to capture the heterogeneity of the broader population, restricting the generalizability of the results. Larger, multi-center studies are necessary to validate the observed neural patterns across different stages of PD. Second, variations in medication regimens among participants were not controlled for, potentially confounding the effects on functional connectivity and hemodynamic responses. Future research should include detailed medication controls to clarify any medication-related influences on the observed neural patterns. Lastly, the Stroop task typically requires motor responses via button presses, which may activate brain regions beyond those directly measured. Incorporating additional imaging modalities (e.g., fMRI) could provide a more comprehensive understanding of the brain networks involved in PD-related cognitive impairments.

## 6 Conclusion

This fNIRS study examined cortical activation and connectivity changes across different stages of cognitive impairment in PD during the Stroop task. The results observed frontoparietal hypoactivation and increased FC in the left S1 in PD-MCI patients, suggesting early compensatory mechanisms as cognitive function declines from PD-NC to PD-MCI. In contrast, PDD patients observed reduced interhemispheric and intrahemispheric FC, suggesting diminished capacities to recruit frontoparietal networks for cognitive task performance. These findings provide an understanding of progressive changes in brain networks involved in cognitive functions across various PD stages, highlighting the potential of fNIRS for assessing neural changes associated with cognitive decline in PD.

## Data Availability

The dataset used in this study is subject to strict privacy and ethical protection guidelines. Access to the data is restricted to authorized personnel only, and all data is anonymized to ensure the confidentiality of participants. Ethical approval was obtained from the Ethics Committee of Sichuan Ba Yi Rehabilitation Center (Sichuan Rehabilitation Hospital), and the study adheres to all relevant ethical standards and data protection regulations. No personal identifiers are included in the dataset to maintain participant privacy. Requests to access the datasets should be directed to Yulai Gong, gongyulai990609@163.com.
