# Supplementary material for: Stage-specific functional networks associated with cognitive impairment in Parkinson's disease: a pilot fNIRS study
Source: Front Aging Neurosci. 2025 May 13;17:1562203. doi: 10.3389/fnagi.2025.1562203 (PMC12106520; doi:10.3389/fnagi.2025.1562203)
Supplement: Supplementary file 1 [file Supplementary_file_1.docx]

**Supplementary Material**

| **Table 1** Cognitive domains and neuropsychological tests | |
| --- | --- |
| **Cognitive domain** | **Cognitive test** |
| **Memory** | Auditory verbal learning test-delayed recall(Siciliano et al. 2017) |
|  | Rey Osterrieth complex figure-delayed recall(Zhang et al. 2016) |
| **Language** | Boston naming test(Goldman et al. 2015) |
|  | Verbal fluency test(Goldman et al. 2015) |
| **Attention/working memory** | Symbol-Digit Modalities Test(Goldman et al. 2015) |
|  | Trait making test-A(Goldman et al. 2015) |
| **Executive function** | Color Word Test-times(Zhang et al. 2016) |
|  | Color Word Test-right(Zhang et al. 2016) |
|  | Trait making test-B(Goldman et al. 2015) |
| **Visuospatial** | Rey Osterrieth complex figure-copy(Somerville et al., 2000.) |
|  | Clock drawing test(Somerville et al., 2000.) |
